# Efficiency and equity considerations in the preferences of health policy-makers in Israel

**DOI:** 10.1186/s13584-017-0142-7

**Published:** 2017-04-01

**Authors:** Amir Shmueli, Ofra Golan, Francesco Paolucci, Emmanouil Mentzakis

**Affiliations:** 1 Department of Health Management, The Hebrew University-Hadassah School of Public Health, POB 12272, Jerusalem, 91120 Israel; 2 The Center for Academic Studies, Or Yehuda, Israel; 3 School of Management and Governance, Murdoch University, Murdoch, Australia; 4 School of Economics, Management and Statistics, University of Bologna, Bologna, Italy; 5 Economics Department, School of Social Sciences, University of Southampton, Southampton, UK

**Keywords:** Efficiency, Equity, Policy-makers, Health, DCE, 1000minds, Israel

## Abstract

**Background:**

There is a traditional tension in public policy between the maximization of welfare from given resources (efficiency) and considerations related to the distribution of welfare among the population and to social justice (equity). The aim of this paper is to measure the relative weights of the efficiency- and equity-enhancing criteria in the preferences of health policy-makers in Israel, and to compare the Israeli results with those of other countries.

**Methods:**

We used the criteria of efficiency and equity which were adopted in a previous international study, adapted to Israel. The equity criteria, as defined in the international study, are: severity of the disease, age (young vs. elderly), and the extent to which the poor are subsidized. Efficiency is represented by the criteria: the potential number of beneficiaries, the extent of the health benefits to the patient, and the results of economic assessments (cost per QALY gained). We contacted 147 policy-makers, 65 of whom completed the survey (a response rate of 44%). Using Discrete Choice Experiment (DCE) methodology by 1000Minds software, we estimated the relative weights of these seven criteria, and predicted the desirability of technologies characterized by profiles of the criteria.

**Results:**

The overall weight attached to the four efficiency criteria was 46% and that of the three equity criteria was 54%. The most important criteria were “financing of the technology is required so that the poor will be able to receive it” and the level of individual benefit. “The technology is intended to be used by the elderly” criterion appeared as the least important, taking the seventh place. Policy-makers who had experience as members of the Basket Committee appear to prefer efficiency criteria more than those who had never participated in the Basket Committee deliberations. While the efficiency consideration gained preference in most countries studied, Israel is unique in its balance between the weights attached to equity and efficiency considerations by health policy-makers.

**Discussion:**

The study explored the trade-off between efficiency and equity considerations in the preferences of health policy-makers in Israel. The way these declarative preferences have been expressed in actual policy decisions remains to be explored.

## Background

There is a traditional and long-standing tension in economics between efficiency - defined as the maximization of welfare - and equity, which includes considerations of equality, the distribution of welfare and social justice.

In terms of health policy, the aspiration to efficiency is equivalent to the maximization of health. When health is measured as Quality Adjusted Life Years (QALYs), as is the case in economic assessments of health technologies, efficiency is identified with the maximization of QALYs. However, maximization of health itself does not take into account considerations of equality, justice, medical need, etc. [1], and when health policy is concerned with the sick or poor, some efficiency might have to be given up.

Policy-makers try to “conciliate” [2] between efficiency and equity considerations when formulating health policy. However, the trade-offs between efficiency and equity are seldom made explicit, and they are usually dealt with on an “ad hoc” basis. Alan Williams, the eminent British health economist, stated: ‘Health systems typically pursue two broad objectives: to maximize the health of the population served, and to reduce inequalities in health…there is conflict between achievement of these two objectives, so that – in setting policy – an explicit weight should be given to each’ ([3], p. 64).

The purpose of this study is to estimate these weights in the preferences of Israeli health policy-makers and compare them to the results in other countries.

### Making health policy under multi-criteria conditions

Decisions on the allocation of social resources among competing uses in health systems are extremely complex. The amount of resources available to the health system is limited, and cannot possibly satisfy all the wants and needs of the population. The decision area which most sharply and dramatically represents the need for priority setting is that of determining which new technologies will receive public funding (within the package of benefits) and which will not. In this situation, priority setting becomes vital and common in many systems. However, this only serves to emphasize the issue of the necessity to consider several and sometimes-contradictory criteria – in many cases equity and efficiency – in decision- making and formulation of health policy. Economic assessments, e.g. cost effectiveness analysis, a primary tool used for prioritizing new technologies in terms of efficiency, do not take into account the distribution of health gains and healthcare among citizens.

Most of the countries where the package of benefits is funded by public money struggle with the question of how to maintain a formal prioritization process that is not only transparent and evidence-based but also reflects public preferences, at least those of policy-makers. This process should be guided by a presentation of criteria together with their weights, in order to yield efficient, fair and consistent decisions, reflecting public preferences.

An international study (hereinafter “the international study”) has recently begun to explore the importance of different criteria in the decision-making processes in different countries. Results have been collected in these countries: Uganda, Nepal, Brazil, Cuba and Norway [4, 5], as well as Austria [6], Spain [7], China [8] and Hungary [9]. All the countries made use of the DCE methodology, with a similar questionnaire (adapted to each national health system) and with analysis, which follows standardized protocol that allows cross-country comparisons. Six criteria reflected the mix of efficiency and equity considerations in the comparison of various technologies: disease severity, number of potential beneficiaries, age groups of potential beneficiaries, the level of the health benefits enjoyed by the beneficiaries of the technology, the extent of willingness to subsidize the poor, and the cost-effectiveness of the technologies.

Israel faces similar concerns. Two recent Israeli studies report relevant findings. A 2008 study concluded, on the basis of an extensive review of the literature, that three main considerations stand at the basis of the prioritization of new technologies: (1) medical need, appropriateness and clinical benefit (2) efficiency (3) equality, solidarity and other ethical or social values [10].

Another study of 2011 [11] found that the preferences of the policy-makers in Israel were related to the benefit, reduction of inequality, lifesaving and allocation to special populations. A greater importance had been attached to the reduction of inequalities than to life extending (for a short term), and the consideration of benefit was preferred in comparison with life-extending and inequality reduction.

### Objectives

The objective of this study is to analyze the relative importance of efficiency and equity considerations in the preferences of the health policy-makers in Israel at the declarative level, and to compare the Israeli results with the results obtained in the international study.

## Methods

### The questionnaire

The data collection process for Israel followed the same protocol as that of the international study with a Hebrew version of the questionnaire. The questionnaire was adapted to the characteristics of the Israeli system.

Based on literature reviews and focus groups, the international study identified six key criteria used in health policy decisions. Each criterion is measured using “levels” (see below for more details). The criteria and their levels were:

1. Disease severity – measured by healthy life expectancy (2 levels – more than 2 years or less)
2. Total beneficiaries – number of affected patients who could potentially benefit (2 levels – more than 100,000 or less)
3. Age – target age groups (3 levels - young, middle, and old ages)
4. Individual benefits (2 levels – more than 5 years in full health or less),
5. Willingness to subsidize the poor (2 levels – greater than 70% government subsidization or less),
6. Cost-effectiveness (2 levels - cost per QALY gained more than Gross National Product per capita or less).

Albeit “willingness to subsidize the poor” is used in other countries, this criterion is not relevant in Israel, where a National Health Insurance system operates and funds all technologies included in the national package of health benefits (apart from a small copayment). Hence, this criterion was defined as: “The funding of the technology is necessary so that the poor will also be able to receive it” – yes or no. This definition was accompanied by the clarification that it is intended to reflect situations where the cost of the technology to the individual is relatively low such that most of the population would be able to purchase it either out of pocket or via complementary or private insurance policies, but the poor would not be able to use it without public funding. For example, the actual copayment in infant vaccination or pregnancy screening tests in Israel is relatively high and many poor families refrain from using these services.

The age criterion was adjusted to provide clarity to the respondents and was split into two separate criteria representing mutually exclusive age groups: “The technology is intended to treat a disease common primarily among children” – yes or no, and “the technology is intended to treat a disease common primarily among the elderly” – yes or no. These two criteria do not appear as “yes” for both for the same technology, but could appear as “no” for both, meaning that the technology is intended to treat a disease common primarily among middle- aged patients, or a disease which is not age related.

### Criteria classification

Following the international study, equity criteria were defined as those dealing with the distributional impact across subpopulations, and included: disease severity, age (including all the age groups), and willingness to subsidize the poor (as adapted to Israel). Efficiency criteria included: the potential number of beneficiaries, the health benefit to the individual patient and the results of the economic assessment (cost per QALY).

We note that the preference of a technology intended primarily for children over one intended primarily for the elderly might express (also) an efficiency consideration, since the treatment of a child yields more life years (in better health) than treatment given to an elderly person. Below we used the two classifications of the age criteria.

### Estimation

DCE, also called Conjoint Analysis, is a declarative method which makes use of a questionnaire which details various combinations of the attributes’ (criteria) levels to measure the preferences and the relative importance assigned to each criterion [12].

The method is based on repetitive selections between pairs of technologies by various combinations of the criteria levels. A series of pairs is presented to the respondent, where each one includes two different scenarios (combinations of the various levels of each of the criteria) and the respondent selects which of the scenarios (“technologies”) she prefers.

The analysis in the international study used the conditional logit model. The importance of the criterion is reflected by its estimated coefficient in a regression where the dependent variable is the probability of selection one technology over the other. For any profile of the criteria (i.e., a set of criteria levels), the predicted probability of selection can be calculated. In this study, the conjoint analysis was conducted using the internet-based software “1000Minds”, a software used for prioritization and ranking. The software uses a unique method for deriving weights, known as PAPRIKA (‘Potentially All Pairwise RanKings of all possible Alternatives’) [13]. For any profile of the criteria, a predicted desirability (scored 0–100 or 0–1) can be calculated.

Because of the transitivity property used by the software, the number of questions that every participant has to answer varies based on the participant’s responses. In this survey, which includes 7 criteria (recall that the age criterion had been decomposed into two criteria), each of which is defined with 2 levels, an average of 12 questions were required in order to calculate the weights of the criteria.

A small pilot involving five policy-makers was conducted and the questionnaire was revised based on their comments.

### The Sample

The sample was compiled from the distribution list of the National Institute for Health Policy Research and the invitation list to the last Dead Sea Conference (December 2013), which gathers Israeli health policy-makers to discuss issues related to Israeli health policy. It included past and present senior managers from the Ministry of Health, Ministry of Finance, sickness funds, Israeli Medical Association, and hospital directors.

### Data collection process

The questionnaire was administered online inviting participants through email. An explanation sheet introduced the survey along with detailed definitions of each of the criteria, as described above. Following the survey, participants were requested to provide some demographic information. In total, out of the 147 policy-makers contacted, 65 completed the choice experiment (a response rate of 44%). Because of technical difficulties related to the possibility of opening Google documents discovered later, only 40 provided full demographic details.

Of those participants who completed the survey: 22 were from the sickness funds, 8 from the Health Ministry, either currently or in the past, 15 were hospital directors (2 of whom had previously served in key Health Ministry positions), 4 were past chairmen of the Public Committee for the Determination of the Package of Benefits (the “Basket Committee”), 1 from the Finance Ministry, 2 from the Israel Medical Association and 2 from the management of the National Institute for Health Policy Research.

### International comparisons

The preferences of the Israeli health policy-makers were compared with those of the policymakers in the countries of the international study. Given the different DCE method implemented in the present study, the comparison consisted of the following components: First, the relative ranking of the criteria was compared. Second, the predicted Israeli ranking was obtained for a sample of *hypothetical* technologies (taken from [4]). Three hypothetical technologies were defined: S0 – a technology where all the criteria are at a high level (“yes”), S1 – a technology where all the equity criteria are at a high level and the efficiency criteria at a low level, and S2 – a technology where all the efficiency criteria are at a high level and the equity criteria at a low level. This comparison was performed by calculating the relative desirability of technologies S2 and S1 with respect to technology S0. These were compared with the predicted probabilities of selection in the international study.

We also calculated the ranking of an additional technology (S1.1) which is intended primarily for children, as well as of a technology (S1.2) which is intended both for children and for the elderly.

Third, the predicted Israeli ranking of a sample of *actual* health technologies was obtained and compared to that obtained in Austria and Spain. The characteristics of the technologies were taken from the Austrian study.

## Results

### The importance of the efficiency and equity considerations in Israel

Table 1 presents the mean weights of the different criteria, as derived from the survey. The most important criteria are “funding the technology is necessary so that the poor will also be able to receive it”, clearly an equity criterion, and “benefit to the individual”, a significant efficiency criteria. The third ranked criterion is also an efficiency criterion “the number of patients requiring the technology”. The fourth is an equity criterion (as per the international study) or an efficiency criterion (by the alternative definition) which specifies whether the technology was intended mainly for children. The criterion regarding whether the technology was intended primarily for the elderly is, however, the least important, taking the seventh place. The fifth criterion is “cost per QALY” which is the most significant efficiency criterion, and the sixth is the criterion “the technology is intended for patients suffering from a serious disease”, an equity criterion.

**Table 1:** The weights of the criteria in Israel

	Number of respondents	The technology is intended for patients suffering from a severe disease	Funding the technology is required so that the poor can receive it	The Technology is intended to treat a disease common among children	The technology is intended to treat a disease common among the elderly	Individual Benefit	The number of patients requiring the technology	Cost per QALY	Total
Type of criteria		equity	equity	equity	equity	efficiency	efficiency	efficiency	
Total respondents	65	11%	19%	14%	10%	19%	15%	12%	100%
Rank		6	1	4	7	1	3	5	
International study definitions	Equity weight = 54%		Efficiency weight = 46%						100%
Alternative definition^a^	Equity weight = 40%		Efficiency weight = 60%						100%

^a^Seeing the criteria “the technology is targeted to children” as an efficiency criterion

In total, according to the international study’s classification, the efficiency criteria comprise a total weight of 46% and the equity criteria – 54%. According to the alternative definition (preference of technologies intended for children seen as an efficiency criteria), the equity criteria weight drops to 40% and the efficiency criteria weight rises to 60%.

### The importance of efficiency and equity considerations in selected groups of the Israeli health policy-makers

The 40 respondents who responded to the demographic survey (and could be assigned to specific subgroups) assigned somewhat different weights, compared to those who did not, to the two age-criteria: They assigned significantly higher weight to the criterion specifying that the technology is mainly used by children (16% vs. 11%), and lower weight to the criterion specifying that the technology is used mainly by the elderly (9% vs. 12%) (Table 2). The overall weights assigned to the equity and efficiency criteria are similar (Table 3), however.

**Table 2:** The weights of the criteria in selected groups of Israeli health policy-makers

	Number of respondents	The technology is intended for patients suffering from a severe disease	The Number of patients requiring the technology	The Technology is intended to treat a disease common among children	The technology is intended to treat a disease common among the elderly	Individual benefit	Funding the technology is required so that the poor can receive it	Cost per QALY	Total
Type of criterion		equity	efficiency	equity	equity	efficiency	equity	efficiency	
Age above 65	11	9%	16%	16%	10%	17%	19%	12%	100%
Age under 65	29	10%	16%	16%	8%	19%	20%	12%	100%
P*		0.62	0.82	0.75	0.27	0.34	0.68	0.95	
Physician	25	11%	**14%**	16%	9%	18%	20%	12%	100%
Non-physician	15	8%	**18%**	15%	9%	18%	19%	13%	100%
P*		0.19	**0.04**	0.65	0.95	0.98	0.75	0.61	
Served on Basket Committee	11	9%	16%	15%	9%	20%	**16%**	**16%**	100%
Never did	29	10%	16%	16%	9%	17%	**21%**	**11%**	100%
P*		0.57	0.67	0.52	0.87	0.24	**0.04**	**0.03**	
Respondents answering the demographic questionnaire	40	10%	16%	**16%**	**9%**	18%	20%	12%	100%
Respondents not answering the demographic questionnaire	25	13%	13%	**11%**	**12%**	21%	17%	12%	100%
P*		0.17	0.25	**0.00**	**0.02**	0.28	0.37	0.88	
Total	65	11%	15%	14%	10%	19%	19%	12%	100%

*2-tailed *t*-test of equality. Significant differences are in boldface

**Table 3:** The overall weights of the efficiency and equity criteria in selected groups of Israeli health policy-makers

	Number of respondents	International study definitions			Alternative definitions^a^		
		Equity weight	Efficiency weight	Total	Equity weight	Efficiency weight	Total
Age above 65	11	55%	45%	100%	38%	62%	100%
Age below 65	29	54%	46%	100%	38%	62%	100%
P*		0.73	0.73		0.89	0.89	
Physician	25	56%	44%	100%	40%	60%	100%
Not physician	15	51%	49%	100%	36%	64%	100%
P*		0.21	0.21		0.26	0.26	
Served on the Basket Committee	11	**49%**	**51%**	100%	**34%**	**66%**	100%
Never did	29	**56%**	**44%**	100%	**40%**	**60%**	100%
P*		**0.03**	**0.03**		**0.04**	**0.04**	
Respondents who answered the demographic questionnaire	40	54%	46%	100%	38%	62%	100%
Respondents who did not answered the demographic questionnaire	25	53%	47%	100%	42%	58%	100%
P*		0.98	0.98		0.07	0.07	
Total	65	54%	46%	100%	40%	60%	100%

*2-tailed *t*-test of equality. Significant differences are in boldface

^a^Seeing the criteria “the technology is targeted to children” as an efficiency criterion

Table 2 also presents the relative importance of the criteria among respondents aged 65+ and younger, physicians and non-physicians, and policy-makers who have ever been members of the public Basket Committee compared to respondents who have never served on that committee. In general, the results indicate that the weights of the criteria are not affected by these characteristics. Two exceptions stand out: First, physicians assign lower weight than non-physicians to the criterion of the number of potential patients – an efficiency consideration (14% vs. 18%). Second, respondents who have experienced the difficulty involved in multi-criteria decision-making and prioritization of technologies during their service as members of the Basket Committee assigned significantly higher importance to the criterion of cost per QALY gained, the main efficiency criterion (16% vs. 11%), and significantly lower importance to the equity criterion regarding the accessibility of the technology to the poor (21% vs. 16%).

Table 3 presents the overall weights of the equity and efficiency considerations in the preferences of various subgroups of policy-makers, according to two classifications of equity and efficiency. While there is no difference in the weight of equity and efficiency considerations between the age groups and between physicians and non-physicians, significant differences exist between those who have served on the Basket Committee and those who have not. Respondents who have participated in the Basket Committee assigned higher weights to efficiency considerations (52% according to the international study’s definition and 66% according to the alternative definition) than other respondents (44% and 60% respectively).

### International comparisons of health policy-makers’ preferences

The Israeli sample (65 respondents) was relatively large, as were the samples in studies conducted in Nepal (66), Spain (69), Austria (70), China (78) and Brazil (73). In the other countries the samples were smaller: 32–34 participants in Norway, 37 in Cuba, 52 in Hungary, and 17 in Uganda. Unlike the studies conducted in Austria, Spain and Norway, where the participants also included academics, in the Israeli study, all the participants were health policy-makers. From an experience standpoint, the vast majority of the participants in this study (90% of the participants in the demographic study) had 10 or more years of experience. This is comparable to the samples of studies in Spain, Norway [5], and Uganda, where only professionals with 10 or more years of experience participated in the study.

### Comparison of the criteria ranking

As the methodology used in the Israeli study differs from that used in the other countries, a direct comparison of the weights assigned to the different criteria was not possible. Instead, we ranked the criteria by their weight (Israel) or effect (elsewhere). The criteria rankings are presented in Table 4 (based on [4–7, 12, 13]).

**Table 4:** Criteria ranking – comparison between countries^a^

Criteria	Israel	Brazil	Cuba	Uganda	Nepal	Norway	Austria	Spain	Hungary	China
Disease severity	6	4	6	2	3	4	5	5	4	5
Number of patients	3	2	1	4	5	6	4	4	7	3
Individual benefit	1	3	5	6	2	3	1	2	1	4
Subsidy to the poor	1	6	3	5	7	7	7	6	5	7
Cost effectiveness	5	1	7	3	4	2	3	1	3	1
Intended for middle-age vs. children^b^	4	5	2	7	6	5	6	7	6	2
Intended for the elderly vs. children^c^	7	7	4	1	1	1	2	3	2	6
Rank correlation with Israel^d^	1	0.20	0.36	**−0.79**	−0.52	−0.65	−0.18	−0.14	−0.18	−0.05
P*		0.66	0.42	**0.04**	0.22	0.10	0.70	0.76	0.70	0.91

*T-test of the hypothesis that the rank correlation = 0. Significant correlations are in boldface

^a^Whenever interactions appeared in the model, the ranking was based on the mean effect

^b^Israel: children vs. other

^c^Israel: elderly vs. other

^d^Adjusted for ties

In most of the countries, the efficiency considerations out-rank the equity considerations. In Israel the findings point to a balance between the equity and efficiency considerations which are alternately ranked. For example, the cost per QALY criterion, a prominent efficiency one, was ranked fifth in Israel, but was ranked in one of the three first places in Brazil, Uganda, Norway, Austria, Spain, Hungary and China. On the other hand, the subsidizing the poor criteria, a significant equity one, was ranked first in Israel, but as last in Nepal, Austria, Norway and China, and as sixth in Brazil and Spain.

Overall, the Israeli ranking is not in accordance (rank correlations are not different from zero) with the rankings in all countries but Uganda and Norway where negative correlations were found.

### Comparison of the ranking of hypothetical technologies

The Israeli prioritization of the hypothetical technologies S0, S1, S1.1, S2 and S1.2 is presented in Table 5. The comparison between Israel and other countries is given in Table 6.

**Table 5:** The Israeli prioritization of the hypothetical technologies

	C R I T E R I A								
Technology	Intended for patients with a severe disease	Number of patients requiring the technology	Intended to treat diseases common to children	Intended to treat diseases common to the elderly	Individual benefit	Requires funding so that the poor can use it	Cost per QALY	Rank	Desirability score
S0	Yes	>100,000	No	Yes	Addition of more than 5 full health years	Yes	Less than per capita GNP	1^st^	0.86
S1.2	Yes	<100,000	Yes	Yes	Addition of less than 5 full health years	Yes	More than per capita GNP	2^nd^	0.54
S2	No	>100,000	No	No	Addition of more than 5 full health years	No	Less than per capita GNP	3^rd^	0.46
S1.1	Yes	<100,000	Yes	No	Addition of less than 5 full health years	Yes	More than per capita GNP	4^th^	0.44
S1	Yes	<100,000	No	Yes	Addition of less than 5 full health years	Yes	More than per capita GNP	5th	0.40

**Table 6:** Prioritization of the hypothetical technologies – comparison between countries (base = S0)

Technology	Israel		Brazil		Cuba		Uganda		Norway		Nepal	
	Desirability Score	Difference in desirability (%)	Prioritization probability	Difference in probability (%)	Prioritization probability	Difference in probability (%)	Prioritization probability	Difference in probability (%)	Prioritization probability	Difference in probability (%)	Prioritization probability	Difference in probability (%)
S0	0.86		0.56		0.16		0.4		0.41		0.4	
S1	0.4	−53	0.04	−93	0.51	213	0.15	−63	0.06	−84	0.08	−79
S2	0.46	−47	0.37	−34	0.08	−52	0.33	−18	0.45	10	0.43	6

The main finding of this comparison is again the balance between the equity and efficiency considerations in Israel: The gap between the desirability score assigned to technology S1, which is entirely pro-equity, and the score of technology S2, which is entirely pro-efficiency, is only 6 percentage-points in favour of efficiency.

In all the other countries [4], the gaps are significantly larger. In all the countries with the exception of Cuba the above gaps are in favour of the efficiency considerations (technology S2).

In Norway and Nepal the preference for technology S2 is greater than that for the base technology (S0). In Cuba, however, the preference for technology S1 is high compared to the base technology (S0).

### Comparison of the rankings of selected actual technologies

The results of this comparison are presented in Table 7. Examination of the table reveals a relatively high correlation among the rankings found in Israel, Austria and Spain.

**Table 7:**
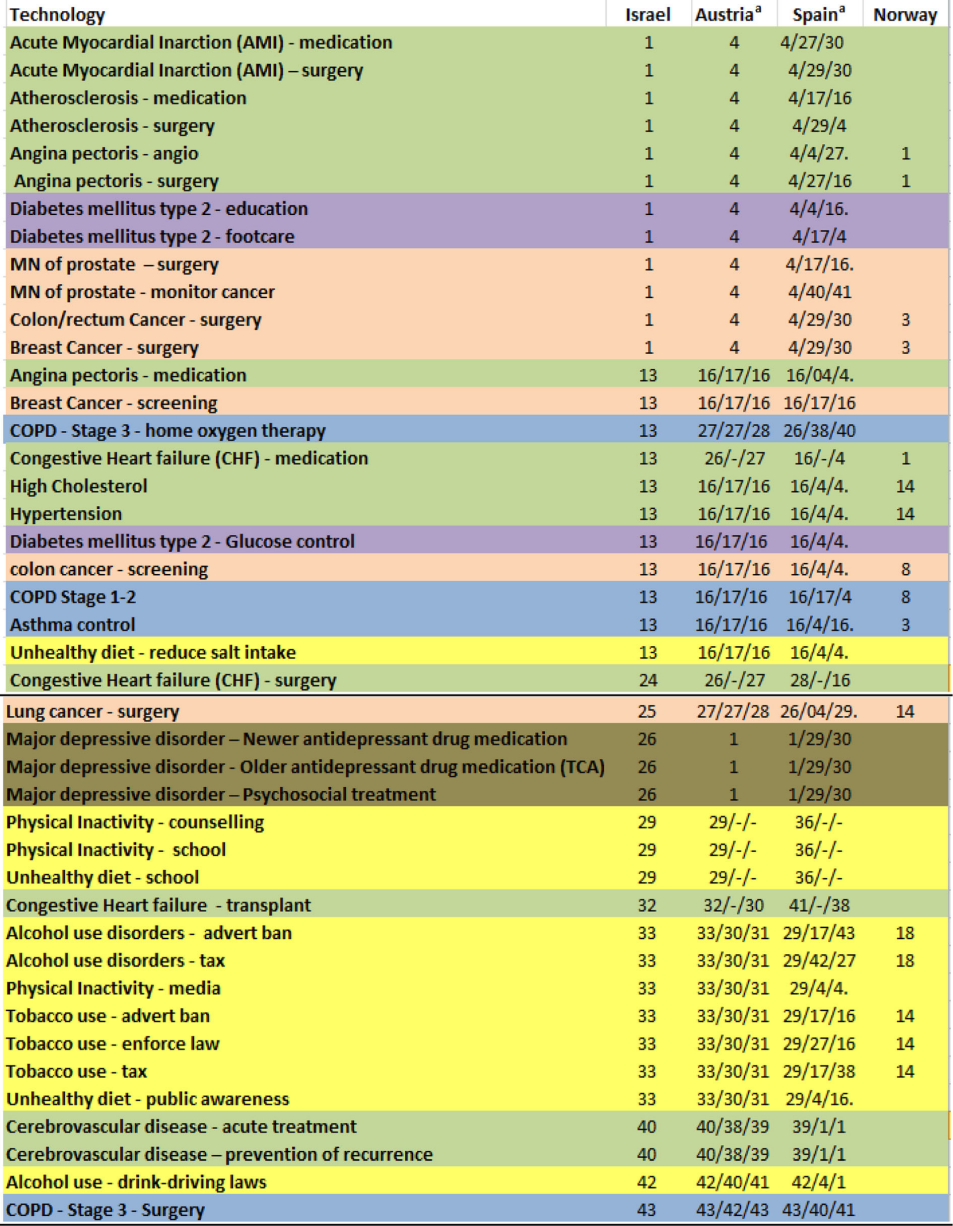
Comparison of the ranking of actual technologies used in the international study^b^

^a^The rankings are presented for three age groups, from left to right – young, middle-age, elderly

^b^Green for cardio-vascular diseases, purple for diabetes, light brown for oncological diseases, blue for respiratory diseases and grey for psychiatric disorders

The technologies that were ranked in first place in the Israeli survey appear at the head of the table. Those technologies were ranked in fourth place in Austria and Spain, after the three psychiatric technologies which were ranked in first place. These, in turn, were ranked only in the 26^th^ place in Israel. However, examination of the data reveals that the psychiatric technologies were the only ones originally classified as “no” in the criterion of willingness to subsidize the poor. As a result, these technologies “lost” 19% (the weight assigned to this criterion by the Israeli survey participants), so the overall weight assigned to them was 57%. Notwithstanding this result – which is based, it would seem, on the lack of an adequate allocation for the treatment of psychiatric disorders in high-income countries (“At present the prospects of an increasing burden of disease for mental disorders are not matched by adequate mental health care spending in high-income countries …” [6]) – the psychiatric technologies would have received 76% in the Israeli study, a weight identical to the technologies that were in first place.

The next group includes 11 technologies, which were ranked according to the results of the Israeli survey in 13^th^ place. All of them were characterized identically in the Austrian data and were ranked in the Austrian and Spanish studies in 16^th^ place, with the exception of two (one in the Spanish study).

An interesting finding in Table 7 is the full correspondence between the Israeli ranking and the Austrian ranking (in the young group), of the 15 technologies at the bottom of the table (starting from 29^th^ place).

## Discussion

In general, approximately equal weight is assigned to the efficiency and equity considerations in the preference of the Israeli health policy-makers participating in the survey. This equality is prominent in the selection of the two most important criteria – individual benefit and the concern for the poor – whose weights were identical (19%).

Comparison of these findings with a survey of Israeli policy-makers which was conducted in 2011 [11]^1^ using general questions about the importance of various criteria reveals consistency in the preferences of the policy-makers in Israel.

While there is no difference in the weights of the equity and efficiency considerations by age and profession (physicians vs. non-physicians) policy-makers who have ever served on the Basket Committee appear to have a higher preference for efficiency considerations than respondents who have not served on that committee.

The comparison of the preferences of Israeli health policy-makers with those of their colleagues in other countries shows that Israel is unique in the balance in the weights assigned to equity and efficiency considerations. In comparison, in most other countries there is a significant preference for efficiency considerations over equity considerations. This gap becomes clearer if we focus on the developed countries included in the international study (Norway, Austria and Spain), where the preference for efficiency over equity is stronger. Israeli preferences agree more with the preferences found in Brazil and Cuba.

## Conclusions

In health systems around the developed world, pressure is increasing for the creation of guidelines and instructions for policy-makers, so that their decisions will be transparent and clear with regard to the criteria used, and will conform to society’s values regarding efficiency and equity. Israel is no exception to this. The process of adoption of new technologies in Israel is well structured; however guidelines and recommendations regarding decision-making itself are missing. For this reason, there is significant variation in the nature of the decisions over the years, both regarding the positions of the individual members of the Basket Committee in any given year, and in the positions of different committees regarding given technologies over time. Israeli health policy at large suffers from similar inconsistencies and lack of transparency.

The present study provided an assessment of the weights assigned by Israeli policy-makers to equity and efficiency considerations in their preferences, compared with their peers abroad. Further research will explore how these weights - obtained on the declarative level – match the actual prioritization of technologies performed by the Israeli Basket Committee.

## Abbreviations

DCE: Discreet Choice Experiment
QALYs: Quality Adjusted Life Years
PAPRIKA: Potentially All Pairwise RanKings of all possible Alternatives

## Footnotes

1 Based on the final report which was submitted to the National Institute for Health Policy Research, as was given to us by courtesy of the researchers and of the National Institute.
